# Long-term outcomes after stress echocardiography in real-world practice: a 5-year follow-up of the UK EVAREST study

**DOI:** 10.1093/ehjci/jeae291

**Published:** 2024-11-12

**Authors:** William Woodward, Casey L Johnson, Samuel Krasner, Jamie O’Driscoll, Annabelle McCourt, Cameron Dockerill, Katrin Balkhausen, Badrinathan Chandrasekaran, Soroosh Firoozan, Attila Kardos, Nikant Sabharwal, Rizwan Sarwar, Roxy Senior, Rajan Sharma, Kenneth Wong, Daniel X Augustine, Paul Leeson

**Affiliations:** Cardiovascular Clinical Research Facility, Division of Cardiovascular Medicine, University of Oxford, John Radcliffe Hospital, Headley Way, Oxford OX3 9DU, UK; Barts and the London School of Medicine and Dentistry, Queen Mary University of London, London, UK; Cardiovascular Clinical Research Facility, Division of Cardiovascular Medicine, University of Oxford, John Radcliffe Hospital, Headley Way, Oxford OX3 9DU, UK; Cardiovascular Clinical Research Facility, Division of Cardiovascular Medicine, University of Oxford, John Radcliffe Hospital, Headley Way, Oxford OX3 9DU, UK; Department of Cardiology, St George’s University Hospitals NHS Foundation Trust, London, UK; Diabetes Research Centre, College of Life Sciences, University of Leicester, Leicester, UK; Cardiovascular Clinical Research Facility, Division of Cardiovascular Medicine, University of Oxford, John Radcliffe Hospital, Headley Way, Oxford OX3 9DU, UK; Cardiovascular Clinical Research Facility, Division of Cardiovascular Medicine, University of Oxford, John Radcliffe Hospital, Headley Way, Oxford OX3 9DU, UK; Division of Imaging Sciences and Biomedical Engineering, Kings College London, London, UK; Department of Cardiology, Royal Berkshire Hospital NHS Foundation Trust, Reading, UK; Wiltshire Cardiac Centre, Great Western Hospitals NHS Foundation Trust, Swindon, UK; Department of Cardiology, Wycombe Hospital, Buckingham Healthcare NHS Trust, Wycombe, UK; Department of Cardiology, Milton Keynes University Hospital NHS Foundation Trust, Milton Keynes, UK; Faculty of Medicine and Health Science, University of Buckingham, Buckingham, UK; Oxford Heart Centre, Oxford University Hospitals NHS Foundation Trust, Oxford, UK; Oxford Heart Centre, Oxford University Hospitals NHS Foundation Trust, Oxford, UK; Department of Cardiology, Northwick Park Hospital, London North West University Healthcare NHS Trust, London, UK; Department of Cardiology, Royal Brompton Hospital, Guy’s and St Thomas’ NHS Foundation Trust, London, UK; Department of Cardiology, St George’s University Hospitals NHS Foundation Trust, London, UK; Lancashire Cardiac Centre, Blackpool Teaching Hospitals NHS Foundation Trust, Blackpool, UK; Liverpool Centre for Cardiovascular Science, University of Liverpool, Liverpool, UK; Department of Cardiology, Royal United Hospitals Bath NHS Foundation Trust, Bath, UK; Cardiovascular Clinical Research Facility, Division of Cardiovascular Medicine, University of Oxford, John Radcliffe Hospital, Headley Way, Oxford OX3 9DU, UK

**Keywords:** stress echocardiography, ischaemic heart disease, long-term outcome

## Abstract

**Aims:**

Stress echocardiography is widely used to assess patients with chest pain. The clinical value of a positive or negative test result to inform on likely longer-term outcomes when applied in real-world practice across a healthcare system has not been previously reported.

**Methods and results:**

Five thousand five hundred and three patients recruited across 32 UK NHS hospitals between 2018 and 2022, participating in the EVAREST/BSE-NSTEP prospective cohort study, with data on medical outcomes up to 2023 available from NHS England were included in the analysis. Stress echocardiography results were related to outcomes, including death, procedures, hospital admissions, and relevant cardiovascular diagnoses, based on Kaplan–Meier analysis and Cox proportional hazard ratios (HRs). Median follow-up was 829 days (interquartile range 224–1434). A positive stress echocardiogram was associated with a greater risk of myocardial infarction [HR 2.71, 95% confidence interval (CI) 1.73–4.24, *P* < 0.001] and a composite endpoint of cardiac-related mortality and myocardial infarction (HR 2.03, 95% CI 1.41–2.93, *P* < 0.001). Hazard ratios increased with ischaemic burden. A negative stress echocardiogram identified an event-free ‘warranty period’ of at least 5 years in patients with no prior history of coronary artery disease and 4 years for those with disease.

**Conclusion:**

In real-world practice, the degree of myocardial ischaemia recorded by clinicians at stress echocardiography correctly categorizes risk of future events over the next 5 years. Reporting a stress echocardiogram as negative correctly identifies patients with no greater than a background risk of cardiovascular events over a similar time period.

## Graphical Abstract

**Graphical Abstract:**
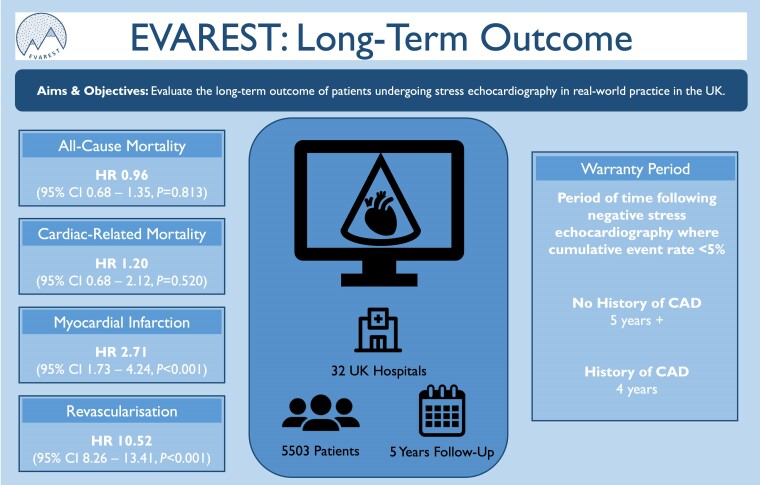

## Introduction

Stress echocardiography is a widely used functional imaging investigation in the diagnosis and management of coronary artery disease (CAD). Early in the development of the practice of stress echocardiography, studies from large centres, and subsequent meta-analyses, demonstrated high levels of accuracy for identification of disease on subsequent coronary angiography.^1,2^ This body of evidence has led to stress echocardiography being recommended as one of the first-line options for investigation of stable chest pain by both the European Society of Cardiology^3^ and American College of Cardiology/American Heart Association.^4^ Stress echocardiography is now in widespread use in clinical practice around the world and over the last two decades has become the most widely available functional imaging test for chest pain within the UK National Health Service (NHS).^5^

To perform stress echocardiograms, operators need expert training to learn how to make qualitative decisions on subtle changes in myocardial wall motion.^6,7^ Whether test accuracy is being maintained when operators apply their experience in real-world practice, across multiple hospitals, with different levels of user expertise, is not well described. How imaging use impacts real-world patient outcomes helps evaluate the clinical value of tests and innovations.^8^ The Echocardiography: Value and Accuracy at Rest and Stress (EVAREST) study provides large-scale, prospective data on echocardiography practice and accuracy across multiple hospitals within the UK healthcare systems.^9^ Data from the study have previously demonstrated high levels of accuracy for stress echocardiography over short timeframes related to findings from coronary angiography.^9^ EVAREST data are now linked with routinely collected healthcare data from NHS England. We have used this combined dataset to understand how confidently the interpretation made at the time of stress echocardiography can be used to inform the patient about their likely long-term outcome.

## Methods

### Patient recruitment and study protocol

The EVAREST study, which includes the British Society of Echocardiography National Review of Stress Echocardiography Practice (BSE-NSTEP), is a multicentre observational study examining the use, performance, and accuracy of stress echocardiography in real-world practice. Study design has been previously described^9^ and registered (NCT03674255). Ethical approval was granted by the Health Research Authority NRES South Central—Berkshire Committee (IRAS: 14/SC/1437). All patients completed written informed consent, and the study was conducted in accordance with the Declaration of Helsinki.

Participants were identified from stress echocardiogram waiting lists at 32 NHS hospitals across the UK and were provided participant information leaflets prior to consent. Stress echocardiograms were conducted in accordance with local hospital protocols and interpreted by clinicians on site. All downstream patient management was determined by local clinicians. Baseline patient demographic data and stress echocardiogram protocol were recorded by the local study team onto an electronic database (Castor EDC, Amsterdam, Netherlands).

### Follow-Up

Long-term follow-up data were obtained from NHS England. Patients consented to collection of outcome data for up to 10 years following their stress echocardiogram. Hospital admission data [including date and reason (defined by ICD-10 coding], as well as any procedures undertaken, such as percutaneous coronary intervention (PCI) or coronary artery bypass grafts (CABG) (defined by OPCS 4.10 coding)], were collected from the NHS Hospital Episodes Statistics Admitted Patient Care (HES APC) dataset. Data on subsequent diagnostic imaging [including computed tomography coronary angiography (CTCA) and invasive coronary angiography/PCI] were obtained from the NHS Diagnostic Imaging Dataset (DID). Mortality data (including date and cause of death) were obtained from the Civil Registrations of Death database. Full details of the ICD-10, OPCS 4.10 and NICIP/SNOMED-CT codes used are in the Supplementary data.

### Statistical analysis

Descriptive data are presented as frequencies (percentage) or median [interquartile range (IQR)] with group comparisons made using *χ*^2^ or Mann–Witney tests, as appropriate. Data normality was assessed using a Shapiro–Wilk test. A *P*-value of ≤0.05 was considered significant. Univariate and multivariate logistic regression was used to examine predictors of abnormal stress echocardiography. Outcome differences were examined using Kaplan–Meier survival curves constructed according to stress echocardiogram result (positive or negative for inducible ischaemia) and ischaemic burden (no ischaemia, 1–2 ischaemic segments, 3–4 ischaemic segments, or ≥5 ischaemic segments). Survival curves differences were investigated using a log-rank test. Cox proportional hazards models were used to calculate hazard ratios (HRs) for all-cause mortality, cardiac-related mortality, myocardial infarction (MI), revascularization (PCI and CABG), and a composite endpoint of ‘hard’ events (cardiac-related mortality or MI). Univariable Cox models were calculated for age, male sex, smoking status, hypertension, hypercholesterolaemia, diabetes, previous CAD, resting regional wall motion abnormality (RWMA), stress echocardiogram result, ischaemic burden, and elective revascularization following stress echocardiography. Significant univariable predictors were then included in a multivariable Cox proportional hazards model, to calculate adjusted HRs. An event-free ‘warranty’ period for a negative stress echocardiogram was defined according to previously reported methods^10^ as the period where the cumulative rate of cardiac-related mortality or MI remains less than 5%. Kaplan–Meier curves were constructed for those patients with a negative stress echocardiogram, according to whether they had a previous diagnosis of CAD. The point at which the survival curve crossed the 5% event threshold indicated the end of the warranty period. Statistical analysis was conducted using SPSS version 29 (IBM, Chicago, IL, USA).

## Results

**Figure 1:**
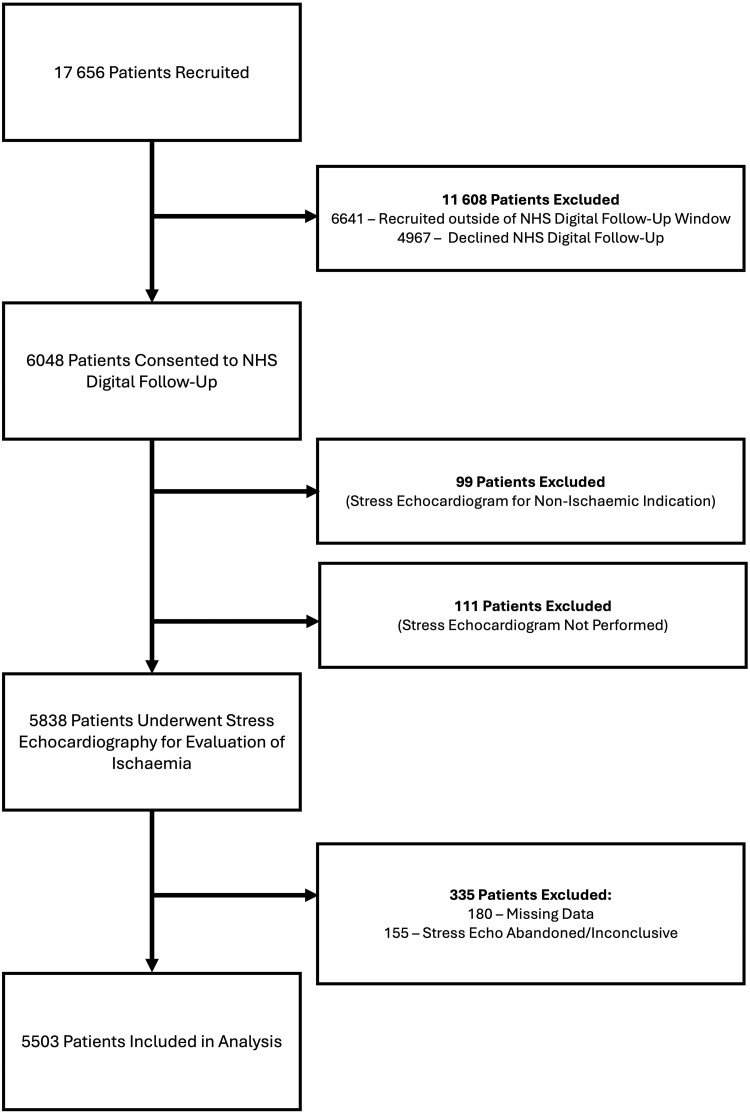
CONSORT diagram showing patient recruitment and reasons for exclusion.

EVAREST recruitment occurred between March 2015 and March 2022. *Figure 1* describes patient inclusion in this analysis. Patients who did not consent to long-term follow-up and had <12 months of follow-up or incomplete data were excluded, leaving 5503 patients for analysis. Information on medical events between April 2018 and March 2023 was obtained with a median follow-up duration of 829 days (IQR 224–1434).

### Patient demographics and stress echo characteristics

*Table 1* presents patient demographics for the analysis dataset. Median age at stress echocardiography was 66 years (IQR 57–74), and 3238 (58.8%) patients were male. Hypertension and hypercholesterolaemia were the most common risk factors, affecting 53.6% and 45.2%, respectively. Pre-existing CAD was present in 33.8% of patients, with 18.3% having a previous MI, 26.9% previous PCI, and 6.8% CABG. As reported in Supplementary data online, *Table S1*, advanced age, male sex, positive smoking history, presence of hypertension, hypercholesterolaemia, diabetes, and previous CABG or RWMA were significant predictors for a positive stress echocardiogram. Dobutamine was the most common stressor (64.0%), then exercise (35.4%, treadmill 27.6%; bicycle ergometer 7.3%), and then pacemaker stress (0.5%). Atropine and left ventricular contrast agents were used in 28.6% and 79.0% of tests, respectively. Of the scans, 14.4% had resting RWMAs, which were significantly associated with positive stress echocardiograms [odds ratio (OR) 3.95; 95% CI 3.27–4.77, *P* < 0.001]. Supplementary data online, *Table S2* reports full stress echocardiogram performance data. Supplementary data online, *Table S3* compares patient demographics and stress echocardiogram characteristics for the dataset to the previously reported EVAREST cohort.^9^ Those who consented to long-term follow-up had a higher risk profile with more male patients and higher rates of hypertension, hypercholesterolaemia, diabetes mellitus, and positive family history of CAD. However, lower rates of prior CAD were present in this group.

**Table 1:** Patient demographics

	Positive SE (*N* = 1050)	Negative SE (*N* = 4453)	*P*-value^a^	Overall (*N* = 5503)
Male (%)	723/1050 (68.9)	2515/4453 (56.5)	**<0**.**001**	3238/5503 (58.8)
Age (years), median (IQR)	68 (60–74)	66 (57–73)	**<0**.**001**	66 (57–74)
BMI (kg/m^2^), median (IQR)	28.1 (25.1–32.1)	28.0 (24.8–31.7)	0.120	28.0 (24.9–31.7)
BSA (m^2^), median (IQR)	1.98 (1.82–2.14)	1.95 (1.78–2.11)	**0**.**001**	1.95 (1.79–2.12)
Smoking status				
Non-smoker (%)	461/1013 (45.5)	2195/4314 (50.9)	**0**.**002**	2656/5327 (48.3)
Ex-smoker (%)	410/1013 (40.5)	1623/4314 (37.6)	0.093	2033/5327 (36.9)
Current smoker (%)	142/1013 (14.0)	496/4314 (11.5)	**0**.**026**	638/5327 (11.6)
Hypertension (%)	650/1030 (63.1)	2301/4314 (53.3)	**<0**.**001**	2951/5344 (53.6)
Hypercholesterolaemia (%)	587/1030 (57.0)	1898/4314 (44.0)	**<0**.**001**	2485/5344 (45.2)
Diabetes mellitus (%)	315/1030 (30.6)	853/4314 (19.8)	**<0**.**001**	1168/5344 (21.2)
Peripheral vascular disease (%)	38/1030 (3.5)	119/4315 (2.6)	0.097	138/5345 (2.5)
Family history of coronary disease (%)	250/1030 (24.3)	954/4314 (22.1)	0.136	1204/5344 (21.9)
Pre-existing CAD (%)	476/1044 (45.6)	1386/4439 (31.2)	**<0**.**001**	1862/5483 (33.8)
Previous MI	266/1041 (25.6)	740/4431 (16.7)	**<0**.**001**	1006/5472 (18.3)
Previous PCI	381/1041 (36.6)	1102/4431 (24.9)	**<0**.**001**	1483/5475 (26.9)
Previous CABG	141/1043 (13.5)	232/4435 (5.2)	**<0**.**001**	373/5478 (6.8)

Presented as no./total no. (percentage).

^a^ *P*-value for comparison between positive and negative stress echocardiogram. Bold values indicate *P* ≤ 0.05.

### Prediction of all-cause and cardiac-related mortality

**Figure 2:**
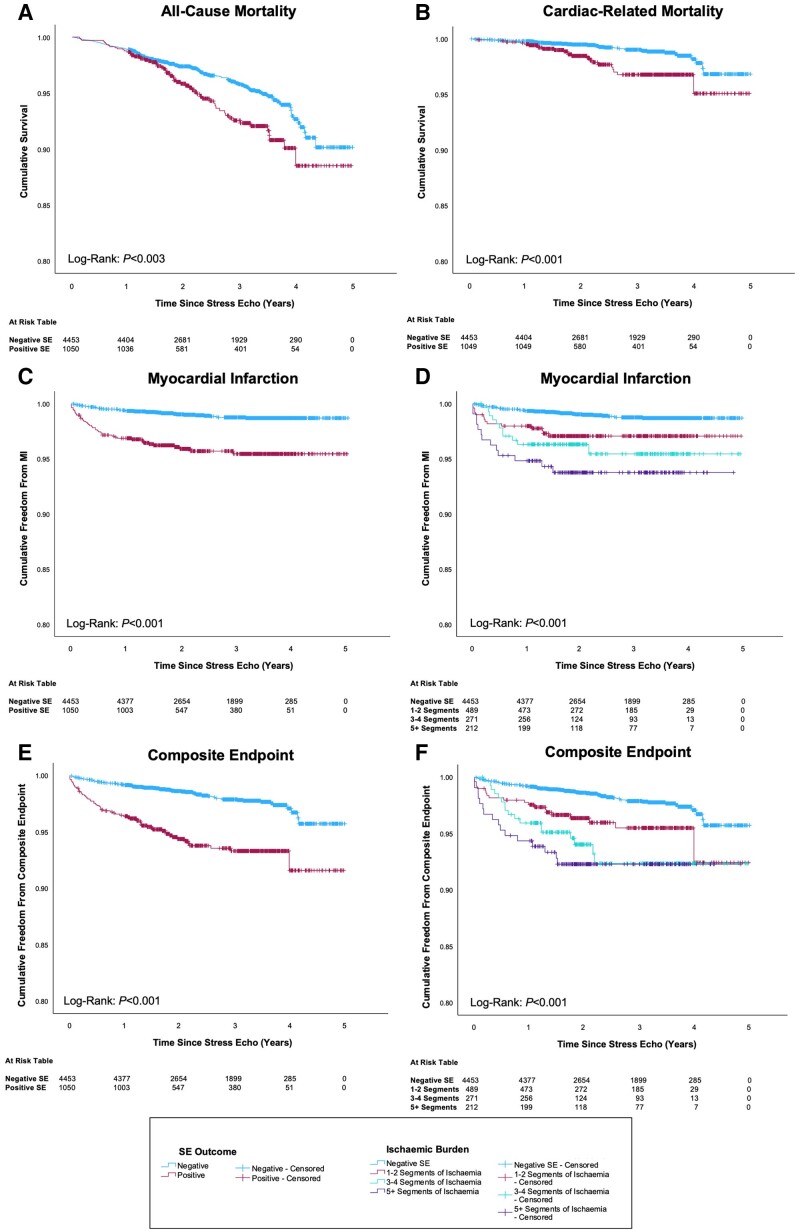
Kaplan–Meier curves for all-cause mortality (*A*), cardiac-related mortality (*B*), MI (*C* and *D*), and composite endpoint of MI or cardiac-related mortality (*E* and *F*). Survival curves in *A–C* and *E* are plotted by stress echocardiogram outcome, whilst *D* and *F* are plotted by ischaemic burden.

Kaplan–Meier survival curves for all-cause and cardiac-related mortality are shown in *Figure 2A* and *B* and demonstrate a higher all-cause mortality (*P* < 0.003) and cardiac-related mortality (*P* < 0.001) in those with a positive stress echocardiogram. During follow-up, 236 patients died from any cause (4.3%). Of these, 61 had a positive stress echocardiogram (*n* = 61/1050, 5.8%) and 175 had a negative stress echocardiogram (*n* = 175/4453, 3.9%). Sixty-four patients (1.2%) died from a cardiac cause: 23 had a positive stress echocardiogram (*n* = 23/1050, 2.2%) and 41 had a negative stress echocardiogram (*n* = 41/4453, 0.9%). In univariable Cox proportional hazards modelling, positive stress echocardiography was a significant predictor of all-cause mortality (HR 1.55; 95% CI 1.16–2.07, *P* = 0.003). However, when adjusted for age, male sex, smoking status, hypertension, diabetes, resting RWMA, and elective revascularization, the association lost significance (HR 0.96; 95% CI 0.68–1.35, *P* = 0.813) (*Table 2*). Similarly, positive stress echocardiography predicted cardiac-related mortality in univariable analysis (HR 2.52; 95% CI 1.51–4.20, *P* < 0.001), but this association was lost after adjustment (HR 1.20; 95% CI 0.68–2.12, *P* = 0.520) (see *Table 2*). Supplementary data online, *Table S4* reports full Cox proportional hazards models for mortality.

**Table 2:** Adjusted HRs by abnormal stress echocardiogram and ischaemic burden

Adjusted HRs												
	Positive stress echo			Ischaemic burden								
				1–2 segments of ischaemia			3–4 segments of ischaemia			5+ segments of ischaemia		
	HR	95% CI	*P*-value	HR	95% CI	*P*-value	HR	95% CI	*P*-value	HR	95% CI	*P*-value
All-cause mortality	0.96	0.68–1.35	0.813									
Cardiac-related mortality	1.20	0.68–2.12	0.520									
MI	2.71	1.73–4.24	**<0**.**001**	2.12	1.15–3.91	**0.017**	2.47	1.22–5.02	**0.012**	4.23	2.23–8.03	**<0.001**
Composite endpoint	2.03	1.41–2.93	**<0**.**001**	1.63	0.98–2.70	0.059	1.95	1.09–3.46	**0.024**	2.77	1.59–4.85	**<0.001**
Revascularization	10.52	8.26–13.41	**<0**.**001**	7.30	5.42–9.83	**<0.001**	9.08	6.49–12.71	**<0.001**	22.79	16.92–30.69	**<0.001**

Univariable Cox proportional hazards modelling was carried out for age, male sex, smoking status, hypertension, hypercholesterolaemia, diabetes, previous CAD, and resting RWMA, with statistically significant univariable predictors included in a multivariable model to calculate adjusted HRs. Bold values indicate *P* ≤ 0.05.

### Prediction of MI

*Figure 2C* demonstrates that a positive stress echocardiogram is significantly associated with an increased incidence of future MI (*P* < 0.001). Furthermore, as burden of ischaemia increases, so does risk of future MI (*Figure 2D*). Ninety-one patients (1.7%) suffered a MI during follow-up. Of these, 43 (4.1%) had a positive stress echocardiogram, whilst 48 (1.1%) had a negative stress echocardiogram. Significant univariable risks for MI included age, male sex, diabetes, previous CAD, resting RWMA, and positive stress echocardiography. When included in a multivariable Cox model, age (HR 1.03; 95% CI 1.01–1.05, *P* = 0.015), diabetes (HR 1.57; 95% CI 1.01–2.44, *P* = 0.045), resting RWMA (HR 1.74; 95% CI 1.07–2.84, *P* = 0.027), and positive stress echocardiography (HR 2.71; 95% CI 1.73–4.24, *P* < 0.001) remained independent predictors (*Table 2*). To examine the hazard of ischaemic burden on MI, a separate multivariable model was created that showed age (HR 1.03; 95% CI 1.01–1.05, *P* = 0.009) and ischaemic burden were significant predictors of MI. Adjusted HRs of 2.12 (95% CI 1.15–3.91, *P* = 0.017), 2.47 (95% CI 1.22–5.02, *P* = 0.012), and 4.23 (95% CI 2.23–8.03, *P* < 0.001) were calculated for 1–2 segments, 3–4 segments, and ≥5 ischaemic segments, respectively (*Table 2*). Supplementary data online, *Table S5* reports full Cox proportional hazards models for MI.

### Composite endpoint

A composite endpoint of MI and cardiac-related mortality was reached by 144 (2.6%) patients. As shown in *Figure 2E*, positive stress echocardiography was significantly associated with the composite endpoint (*P* < 0.001). Of those reaching the composite endpoint, 61 (5.8%) patients had a positive stress echocardiogram [males—48 (6.6%), females—13 (4.0%)], whilst 83 (1.9%) had a negative stress echocardiogram [males—60 (2.4%), females—23 (1.2%)]. As ischaemic burden increased, so did the incidence of composite endpoint (*P* < 0.001) (see *Figure 2F*). Univariable predictors of composite endpoint were age, male sex, smoking status, diabetes, previous CAD, resting RWMA, and positive stress echocardiography. Multivariable analysis showed that advanced age (HR 1.05; 95% CI 1.03–1.06, *P* < 0.001), being a current smoker (HR 1.85; 95% CI 1.10–3.12, *P* = 0.021), diabetes (HR 2.04; 95% CI 1.44–2.87, *P* < 0.001), resting RWMA (HR 1.93; 95% CI 1.30–2.86, *P* = 0.001), and positive stress echocardiography (HR 2.03; 95% CI 1.41–2.93, *P* < 0.001) remained predictors of this endpoint (see *Table 2* and Supplementary data online, *Table S7*). A multivariable Cox model examining ischaemic burden showed that advanced age, being a current smoker, diabetes, resting RWMA, and ischaemic burden were all significant predictors of the composite endpoint. Whilst 1–2 segments of ischaemia was not a statistically significant predictor (HR 1.63; 95% CI 0.98–2.70, *P* = 0.059), 3–4 segments (HR 1.95; 95% CI 1.09–3.46, *P* = 0.024) and ≥5 segments (HR 2.77; 95% CI 1.59–4.85, *P* < 0.001) of ischaemia were significant predictors (see *Table 2*).

### Prediction of revascularization

Revascularization was performed in 366 (6.7%) patients (PCI, 284; CABG, 77; PCI and CABG, 5). There was a difference between Kaplan–Meier curves for revascularization between those with positive and negative stress echocardiograms (*P* < 0.001), with 262 (25.0%) patients undergoing revascularization following a positive stress echocardiogram, compared with 104 (2.3%) with a negative stress echocardiogram (see Supplementary data online, *Figure S1*). Univariable predictors of revascularization included advanced age, male sex, hypertension, hypercholesterolaemia, diabetes, previous CAD, resting RWMA, and positive stress echocardiography. When included in a multivariable model, male sex (HR 1.77; 95% CI 1.37–2.27, *P* < 0.001), diabetes (HR 1.33; 95% CI 1.06–1.66, *P* = 0.015), and positive stress echocardiography (HR 10.52; 95% CI 8.26–13.41, *P* < 0.001) remained independent predictors (see *Table 2* and Supplementary data online, *Table S6*). Furthermore, a separate multivariable analysis showed that ischaemic burden predicted revascularization, with HRs of 7.30 (95% CI 5.42–9.83, *P* < 0.001), 9.08 (95% CI 6.49–12.71, *P* < 0.001), and 22.79 (95% CI 16.92–30.69, *P* < 0.001) calculated for 1–2 segments, 3–4 segments, and ≥5 segments of ischaemia, respectively (see *Table 2* and Supplementary data online, *Table S6*).

### Warranty period

**Figure 3:**
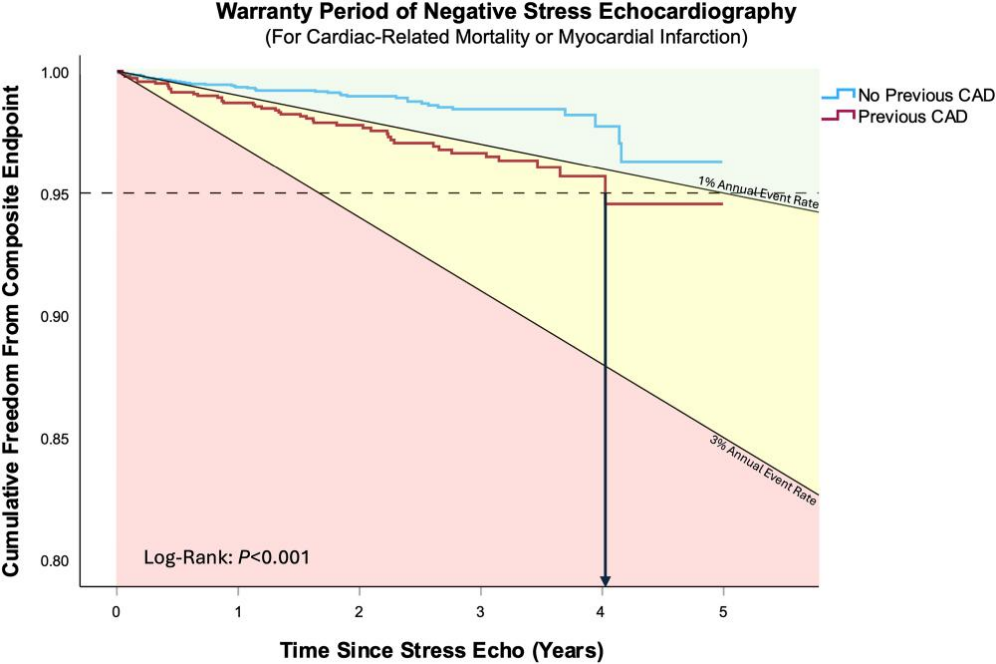
Kaplan–Meier curves for patients with a negative stress echocardiogram by previous CAD diagnosis. Dashed horizontal reference line denotes the 5% event-free threshold which defines the warranty period. Additional reference lines denoting a 1% annual event rate and 3% annual event rate are also plotted.

*Figure 3* shows Kaplan–Meier survival curves for the composite endpoint in patients with a negative stress echocardiogram, constructed according to presence or absence of previous CAD. Of those with a negative stress echocardiogram, 1386 (31.2%) patients had previously diagnosed CAD whilst 3053 (68.8%) patients had no history of CAD. Proportionally fewer cardiac events were observed in those with no previous history of CAD compared with those with a prior diagnosis of CAD (*P* < 0.001), 40 (1.3%) patients vs. 43 (3.1%) patients, respectively. Furthermore, the survival curve for patients without previous CAD did not cross the 5% event threshold, indicating a warranty period of at least 5 years for this group of patients. In addition, the annual adverse event rate remained less than 1% per year (defined as low risk by the ESC guidelines.^3^) The survival curve for patients with a negative stress echocardiogram and a history of CAD crossed the 5% event threshold at 4.02 years, suggesting an event-free warranty period of up to 4 years. Whilst the annual event rate for this group was greater than 1% per year (1.5% per year), this was lower than the 3% per year rate defined as a high-risk population according to the ESC guidelines.

## Discussion

In real-world practice, the degree of myocardial ischaemia recorded by clinicians at stress echocardiography correctly categorizes risk of future cardiovascular events over the next 5 years. Reporting a stress echocardiogram as negative in someone without a history of CAD correctly identifies patients with no greater than a background risk of cardiovascular events over a similar time period. This large-scale prospective study provides contemporary data regarding how effectively stress echocardiography is being interpreted within routine healthcare practice across UK-based NHS hospital networks. Adequate endocardial definition has been shown to lead to higher levels of accuracy.^11^ In this study, 79.0% of patients underwent a contrast-enhanced echocardiogram, which reflects UK practice in stress echocardiography, which may have contributed to ensuring high levels of accuracy in an unselected cohort.

Stress echocardiography is widely used as a first-line investigation in patients presenting with stable chest pain to risk stratify and determine which patients should be referred for coronary angiography.^5,6^ This also means, if the operator decides the stress echocardiogram is negative, the patient will not be offered angiography or medication, which raises a hypothetical risk that they are not being offered an intervention that could reduce their risk of future events. The findings from this study are reassuring that patients identified with a negative stress echocardiogram do indeed fit with a lower risk group with a risk of future cardiovascular events below the general population rate. The counter concern when managing patients with coronary disease is the accurate identification of those at highest risk of MI so that treatment can be intensified. This study shows that the incidence of MI increases following a positive stress echocardiogram, with incidence increasing in a stepwise manner according to how the operator is reporting ischaemic burden based on the number of segments of inducible wall motion abnormalities. These findings are consistent with previous studies that show association between stress echocardiogram classification and rates of both revascularization and MI.^12,13^ It should be noted, however, that the decision to revascularize is likely driven by angiographic findings in the context of a positive stress echocardiogram. Revascularization as an outcome measure is therefore subject to bias and is hence reported as a ‘soft’ outcome, in comparison with the ‘hard’ events of mortality or MI.

Current guidance by the American College of Cardiology states that all negative results from a functional imaging investigation have a warranty period of 1 year, after which the result should be disregarded, whereas CTCA has a warranty period of 2 years.^4^ In this study, those with a negative stress echocardiogram and no prior history of coronary disease have an annual hard event rate of less than 1%, consistent with a low risk of future events. Although those with a negative stress echocardiogram and a history of pre-existing coronary disease are at slightly higher risk, their risk remains less than 1.5% per year. Applying a method previously used for evaluating a ‘warranty period’ following combined CTCA and PET, we have shown that the ‘warranty period’ of stress echocardiography extends for a period of up to 4 years in those with a previous diagnosis of CAD and at least 5 years for those with no previously documented coronary disease. This finding supports reports from single centres that have shown similar event-free periods following negative stress echocardiography.^14–16^ Cortigiani *et al*. showed advanced age and presence of diabetes or use of anti-anginals increase risk despite a negative stress echocardiogram.^15,17^ A 2007 meta-analysis of 3000 patients pooled from multiple studies also showed sex as a determinant of event rate following a negative stress.^18^ Similarly, in our models, male sex was an independent predictor of outcomes, and these findings suggest that, although a negative stress echocardiogram should be reassuring, decisions about risk factor modification still need to take into account the entire risk factor profile of the patient.^19^

Significant differences in all-cause and cardiac survival curves were evident between positive and negative stress echocardiograms. However, in adjusted Cox proportional hazards modelling, the finding of a positive stress echocardiogram was not an independent predictor of either all-cause or cardiac-related mortality. Mortality risk could be identified based just on the more typical risk factors of advanced age, male sex, smoking status, and diabetes, which were more evident in those with a positive stress echocardiogram. This differs from previous studies that have reported positive stress echocardiography is a predictor of mortality.^15,20^ These prior studies were performed between 1985 and 2011. It is likely survival rates have improved over the few decades with significant changes in management, thus potentially diluting predictive power of a positive stress echocardiogram alone.^21,22^ On the other hand, in their recent large study of 3191 patients undergoing dipyridamole stress echocardiography, Gaibazzi *et al*. also found that the presence of inducible ischaemia on stress echocardiography was unable to independently predict risk of future mortality. Stress echocardiography was, however, able to predict risk of future MI and a combined endpoint of MI and all-cause mortality.^13^ These findings are in keeping with our present study. In both these studies, the inability of inducible ischaemia during stress echocardiography to correctly predict future mortality as a standalone endpoint may result from the lower event rates observed in both studies, compared with earlier studies.^15,20^ As mentioned above, this may relate to a lack of power to detect a meaningful difference in the frequency of these events given the reduction in event rate brought about from recent improvements in patient management combined with limited follow-up duration. Interestingly, resting RWMAs remained an independent predictor. This may be because of its role as a marker of previous MI or scarred, arrhythmogenic myocardium, and also as a surrogate marker of heart failure or reduced ejection fraction. Bangalore *et al.*^23^ showed that baseline ejection fraction was a more powerful predictor of cardiac-related mortality than the extent of ischaemia observed during stress echocardiography. Whilst ejection fraction data are not available in the current study, it is likely that those with RWMAs have a reduced ejection fraction. Therefore, given the inherent link between the two, this may provide an explanation for why we found that RWMA was associated with an increase in mortality, in line with other studies.^13,23^

Studies such as PROMISE^24^ and ISCHEMIA^25^ have sought to determine optimal management of patients presenting with angina. In contrast to the findings of PROMISE, ISCHEMIA found that severity of ischaemia detected was not associated with hard events. Thus, the present data provide support for the functional imaging arm of the PROMISE study (which was predominantly myocardial perfusion scintigraphy), albeit using a more widely available^5^ and ionizing-free alternative. A possible reason for the failure of ISCHEMIA to demonstrate outcome benefit may be due to the high proportion of patients with moderate to severe ischaemia, compared with smaller ischaemic burdens; thus, differences in outcome according to ischaemic burden may not have been detected. In contrast to ISCHEMIA, the prognostic value of detection of any degree of inducible ischaemia by stress echocardiography has also been reported by Gaibazzi *et al*.^13^ They demonstrated that even after adjusting for CAD severity, the presence of ischaemia was associated with a combined endpoint of all-cause mortality and MI. This suggests that stress echocardiography provides incremental prognostic data to CAD burden alone. Given this finding, results from any available anatomical imaging (such as CTCA) should be combined with stress echocardiographic findings to ensure that patients receive appropriate follow-up and a tailored management approach

The study has several limitations. Firstly, lack of quantification of angiographic findings in the NHS England data limited our ability to characterize based on severity of coronary disease. Secondly, a portion of patients were recruited after publication of the ISCHEMIA study, which supported the use of medical management in the first instance,^25^ and this may have changed decision-making to reduce the number of angiographic-defined endpoints and revascularizations. Given that pharmacological co-therapy data were not available from NHS England datasets, we were unable to account for medical management approaches in our multivariable regression models. Whilst medical therapies may have had an effect on patient outcome, given the long-standing guidance on their use, it is likely that most patients would have received appropriate medical therapy, if indicated, in addition to undergoing any invasive procedures. Thirdly, since the follow-up data are limited to investigations and procedures performed within the NHS, those conducted outside the NHS have not been captured. Fourthly, the findings can only be used to infer about clinical practice amongst operators and clinicians working within the NHS. There may be important differences in their training and patient care pathways when compared with other healthcare systems that mean these results are not maintained in other geographic areas. Fifthly, the analysis relies on inclusion of patients who consent to participation in a research study. This may explain why the cohort who consented to long-term follow-up had a slightly higher risk factor profile as they were more engaged with understanding their disease. This might have resulted in a higher incidence of events in this cohort and an underestimation of metrics such as the warranty period. Sixthly, to reflect UK-based practice, the method of stress was at the discretion of the performing clinicians. As such, our study almost exclusively includes echocardiograms performed using exercise or dobutamine, and therefore, conclusions related to other stressors, such as dipyridamole (and associated measurements including coronary flow velocity reserve), are lacking. Finally, although the study was designed to cover a range of hospital sizes and locations as well as allow for variation in patient demographics and language restrictions, there may still be some patients who are not engaged with research and are not included in this analysis.

## Conclusion

This study provides contemporary evidence on how effectively stress echocardiography is being applied within real-world practice within the UK NHS. The results indicate that the current approaches operators use to interpret stress echocardiograms provide robust categorization of risk over long timeframes. A negative stress echocardiogram in someone without a prior history of CAD is effectively identifying patients with low annual event rates for at least 5 years following the test. The operators are also effectively categorizing risk based on the number of inducible ischaemic segments identified during the stress study. The wide-ranging multicentre design of the study gives reassurance that stress echocardiography is being performed to a high standard across the UK.

## Supplementary Material

jeae291_Supplementary_Data

## Data Availability

The data underlying this article will be shared on reasonable request to the corresponding author.

## EVAREST Investigator Group

Abraheem Abraheem^17^, Sanjay Banypersad^18^, Sadie Bennett^19^, Henry Boardman^9,11^, Christopher Boos^20^, Sudantha Bulugahapitiya^21^, Jeremy Butts^22^, Duncan Coles^23^, Joanna d'Arcy^11^, Jacob Easaw^16^, Sarah Fairbairn^24^, Haytham Hamdan^25^, Shahnaz Jamil-Copley^26^, Gajen Kanaganayagam^27^, Ioannis Moukas^28^, Tom Mwambingu^29^, Thuraia Nageh^30^, David Oxborough^31^, Antonis Pantazis^32^, Alexandros Papachristidis^33^, Maria Paton^34,35^, Keith Pearce^36^, Ronak Rajani^37^, Muhammad Amer Rasheed^38^, Naveed A. Razvi^39^, Sushma Rekhraj^26^, David P. Ripley^40^, Shaun Robinson^41^, Kathleen Rose^42^, Michaela Scheuermann-Freestone^43^, Rebecca Schofield^44^, Nancy Spagou^45^, Ayyaz Sultan^46^, Apostolos Tsiachristas^47^, Ross Upton^45^, James Willis^16^, Gary Woodward^45^, Spiros Zidros^48^

**Affiliations:**

^17^Department of Cardiology, Tameside and Glossop Integrated Care NHS Foundation Trust, Ashton-under-Lyne, UK.

^18^Department of Cardiology, East Lancashire Hospitals NHS Trust, Burnley, UK.

^19^Department of Cardiology, Heart & Lung Centre, Royal Stoke University Hospital, University Hospitals of North Midlands NHS Foundation Trust, Stoke, UK.

^20^Department of Cardiology, Poole Hospital NHS Foundation Trust, Poole, UK.

^21^Department of Cardiology, Bradford Teaching Hospitals NHS Foundation Trust, Bradford, UK.

^22^Department of Cardiology, Calderdale and Huddersfield NHS Foundation Trust, Calderdale, UK.

^23^Department of Cardiology, Broomfield Hospital, Mid and South Essex NHS Foundation Trust, Broomfield, UK.

^24^Bristol Heart Institute, University Hospitals Bristol and Weston NHS Foundation Trust, Bristol, UK.

^25^Department of Cardiology, Wrightington, Wigan and Leigh NHS Foundation Trust, Wigan, UK.

^26^Department of Cardiology, Nottingham University Hospitals NHS Trust, Nottingham, UK.

^27^Department of Cardiology, Chelsea and Westminster Hospital NHS Foundation Trust, London, UK.

^28^Department of Cardiology, Warrington and Halton Teaching Hospitals NHS Foundation Trust, Warrington, UK.

^29^Department of Cardiology, The Mid Yorkshire Hospitals NHS Trust, Pinderfields, UK.

^30^Department of Cardiology, Southend University Hospital, Mid and South Essex NHS Foundation Trust, Southend-on-Sea, UK.

^31^Research Institute for Sports and Exercise Science, Liverpool John Moores University/Liverpool Centre for Cardiovascular Science, Liverpool, UK.

^32^Department of Cardiology, North Middlesex University Hospital NHS Trust, London, UK.

^33^Department of Cardiology, King's College Hospital NHS Foundation Trust, London, UK.

^34^Department of Cardiology, Leeds General Infirmary, The Leeds Teaching Hospitals NHS Trust, Leeds, UK.

^35^School of Medicine, University of Leeds, Leeds, UK.

^36^Department of Cardiology, Wythenshawe Hospital, Manchester University NHS Foundation Trust, Manchester, UK.

^37^Department of Cardiology, Guy's and St Thomas' NHS Foundation Trust, London, UK.

^38^Department of Cardiology, Yeovil District Hospital, Somerset NHS Foundation Trust, Yeovil, UK.

^39^Department of Cardiology, East Suffolk and North Essex NHS Foundation Trust, Ipswich, UK.

^40^Department of Cardiology, Northumbria Healthcare NHS Foundation Trust, North Tyneside, UK.

^41^Department of Cardiology, Imperial College Healthcare NHS Trust, UK

^42^Department of Cardiology, Northampton General Hospital NHS Trust, Northampton, UK.

^43^Department of Cardiology, Hampshire Hospitals NHS Foundation Trust, Basingstoke, UK.

^44^Department of Cardiology, North West Anglia NHS Foundation Trust, Peterborough, UK.

^45^Ultromics Ltd, Oxford, UK.

^46^Department of Cardiology, Wrightington, Wigan and Leigh NHS Foundation Trust, Wigan, UK.

^47^Health Economic Research Centre, Nuffield Department of Population Health, University of Oxford, Oxford, UK.

^48^Department of Cardiology, Bedford Hospital, Bedfordshire Hospitals NHS Foundation Trust, Bedford, UK.
