# Supplementary material for: Long-term outcomes after stress echocardiography in real-world practice: a 5-year follow-up of the UK EVAREST study
Source: Eur Heart J Cardiovasc Imaging. 2024 Nov 12;26(2):187–96. doi: 10.1093/ehjci/jeae291 (PMC11781832; doi:10.1093/ehjci/jeae291)
Supplement: jeae291_Supplementary_Data [file jeae291_supplementary_data.zip › Supplemental Material.docx]

**SUPPLEMENTAL MATERIAL**

**EVAREST Investigator Group**

Abraheem Abraheem^1^, Daniel Augustine^2, 3^, Katrin Balkhausen^4^, Sanjay Banypersad^5^, Sadie Bennett^6^, Henry Boardman^7, 8^, Christopher Boos^9^, Sudantha Bulugahapitiya^10^, Jeremy Butts^11^, Badrinathan Chandrasekaran^12^, Duncan Coles^13^, Joanna d’Arcy^7^, Cameron Dockerill^14^, Jacob Easaw^2^, Sarah Fairbairn^15^, Soroosh Firoozan^16^, Haytham Hamdan^17^, Shahnaz Jamil-Copley^18^, Gajen Kanaganayagam^19^, Attila Kardos^8, 20^, Annabelle McCourt^14^, Ioannis Moukas^21^, Tom Mwambingu^22^, Thuraia Nageh^23^, Jamie O'Driscoll^24, 25^, David Oxborough^26^, Antonis Pantazis^27^, Alexandros Papachristidis^28^, Maria Paton^29, 30^, Keith Pearce^31^, Ronak Rajani^32^, Muhammad Amer Rasheed^33^, Naveed A. Razvi^34^, Sushma Rekhraj^18^, David P. Ripley^35^, Shaun Robinson^36^, Kathleen Rose^37^, Nikant Sabharwal^7^, Rizwan Sarwar^7^, Michaela Scheuermann-Freestone^38^, Rebecca Schofield^39^, Roxy Senior^40, 41, 42^, Rajan Sharma^24^, Nancy Spagou^43^, Ayyaz Sultan^44^, Apostolos Tsiachristas^45^, Ross Upton^43^, James Willis^2^, Kenneth Wong^46^, Gary Woodward^43^, William Woodward^14^, Spiros Zidros^47^ and Paul Leeson^14^.

**Affiliations:**

^1^Tameside and Glossop Integrated Care NHS Foundation Trust, Ashton-under-Lyne, UK.

^2^Royal United Hospitals NHS Foundation Trust, Bath, UK

^3^Department for Health, University of Bath, UK

^4^Royal Berkshire Hospitals NHS Foundation Trust, Reading, UK.

^5^East Lancashire Hospitals NHS Trust, Burnley, UK.

^6^Heart & Lung Centre, Royal Stoke University Hospital, University Hospitals of North Midlands NHS Foundation Trust, Stoke, UK.

^7^Oxford University Hospitals NHS Foundation Trust, Oxford, UK

^8^Milton Keynes University Hospital NHS Foundation Trust, Milton Keynes, UK.

^9^Poole Hospital NHS Foundation Trust, Poole, UK.

^10^Bradford Teaching Hospitals NHS Foundation Trust, Bradford, UK.

^11^Calderdale and Huddersfield NHS Foundation Trust, Calderdale, UK.

^12^Great Western Hospitals NHS Foundation Trust, Swindon, UK.

^13^Broomfield Hospital, Mid and South Essex NHS Foundation Trust, Broomfield, UK.

^14^Cardiovascular Clinical Research Facility, RDM Division of Cardiovascular Medicine, University of Oxford, Oxford, UK.

^15^Bristol Heart Institute, University Hospitals Bristol and Weston NHS Foundation Trust, Bristol, UK.

^16^Buckinghamshire Healthcare NHS Trust, High Wycombe, UK.

^17^Wrightington, Wigan and Leigh NHS Foundation Trust, Wigan, UK.

^18^Nottingham University Hospitals NHS Trust, Nottingham, UK

^19^Chelsea and Westminster Hospital NHS Foundation Trust, London, UK.

^20^Faculty of Medicine and Health Science, University of Buckingham, Buckingham, UK.

^21^Warrington and Halton Teaching Hospitals NHS Foundation Trust, Warrington, UK.

^22^The Mid Yorkshire Hospitals NHS Trust, Pinderfields, UK

^23^Southend University Hospital, Mid and South Essex NHS Foundation Trust, Southend-on-Sea, UK.

^24^St. George’s University Hospitals NHS Foundation Trust, London, UK.

^25^Diabetes Research Centre, College of Life Sciences, University of Leicester, UK.

^26^Research Institute for Sports and Exercise Science, Liverpool John Moores University/Liverpool Centre for Cardiovascular Science, Liverpool, UK.

^27^North Middlesex University Hospital NHS Trust, London, UK.

^28^King's College Hospital NHS Foundation Trust, London, UK.

^29^Leeds General Infirmary, The Leeds Teaching Hospitals NHS Trust, Leeds, UK.

^30^University of Leeds, Leeds, UK.

^31^Wythenshawe Hospital, Manchester University NHS Foundation Trust, Manchester, UK.

^32^Guy’s and St Thomas’ NHS Foundation Trust, London, UK.

^33^Yeovil District Hospital, Somerset NHS Foundation Trust, Yeovil, UK.

^34^East Suffolk and North Essex NHS Foundation Trust, Ipswich, UK.

^35^Northumbria Healthcare NHS Foundation Trust, North Tyneside, UK.

^36^Imperial College Healthcare NHS Trust, UK

^37^Northampton General Hospital NHS Trust, Northampton, UK.

^38^Hampshire Hospitals NHS Foundation Trust, Basingstoke, UK.

^39^North West Anglia NHS Foundation Trust, Peterborough, UK.

^40^National Heart and Lung Institute, Imperial College London, UK.

^41^Royal Brompton Hospital, Guy’s and St. Thomas’ NHS Foundation Trust, London, UK.

^42^London North West University Healthcare NHS Trust, London, UK.

^43^Ultromics Ltd, Oxford, UK.

^44^Wrightington, Wigan and Leigh NHS Foundation Trust, Wigan, UK.

^45^Health Economic Research Centre, Nuffield Department of Population Health, University of Oxford, Oxford, UK.

^46^Lancashire Cardiac Centre, Blackpool Teaching Hospitals NHS Foundation Trust, Blackpool, UK.

^47^Bedford Hospital, Bedfordshire Hospitals NHS Foundation Trust, Bedford, UK.

**Supplemental Figure 1**

**
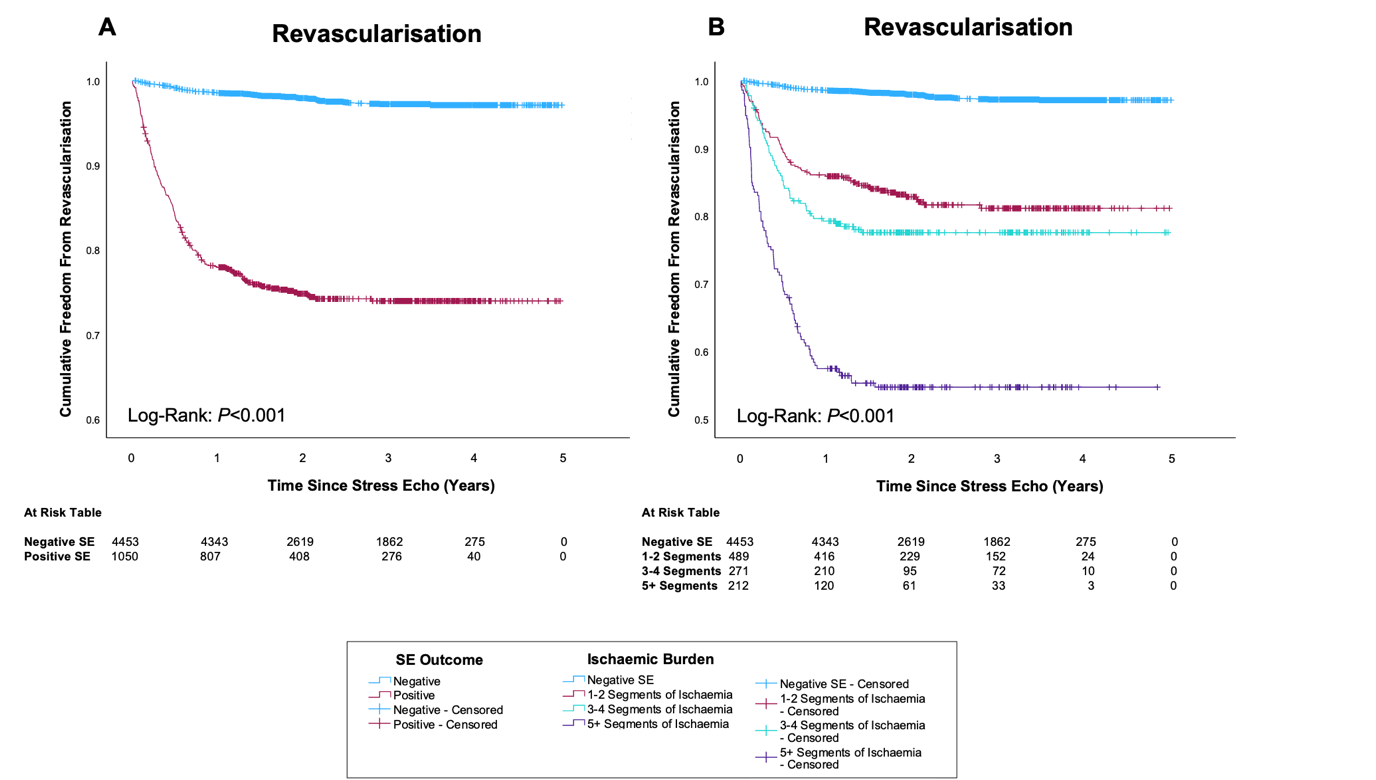
**

**Supplemental Figure 1:** Kaplan-Meier curves examining difference in revascularisation by stress echocardiogram outcome (A) and ischaemic burden (B).

| Supplemental Table 1: Logistic Regression – Predictors of Abnormal Stress Echocardiography | | | | | | | |
| --- | --- | --- | --- | --- | --- | --- | --- |
|  | | Univariate | | | Multivariate | | |
|  | | **OR** | **95% CI** | ***P*-Value** | **OR** | **95% CI** | ***P*-Value** |
| Sex (Male) | | 1.70 | 1.48 – 1.97 | **<0.001** | 1.42 | 1.21 – 1.67 | **<0.001** |
| Age | | 1.02 | 1.01 – 1.02 | **<0.001** | 1.01 | 1.01 – 1.02 | **<0.001** |
| BMI | | 1.00 | 0.99 – 1.02 | 0.494 | - | - | - |
| Smoking Status | |  |  |  |  |  |  |
|  | Ex-Smoker | 1.20 | 1.04 – 1.39 | **0.014** | 1.00 | 0.85 – 1.17 | 0.997 |
|  | Current Smoker | 1.36 | 1.10 – 1.69 | **0.004** | 1.33 | 1.06 – 1.67 | **0.016** |
| Hypertension | | 1.50 | 1.30 – 1.72 | **<0.001** | 1.15 | 0.98 – 1.34 | 0.089 |
| Hypercholesterolaemia | | 1.69 | 1.47 – 1.94 | **<0.001** | 1.38 | 1.19 – 1.61 | **<0.001** |
| Diabetes Mellitus | | 1.79 | 1.54 – 2.08 | **<0.001** | 1.52 | 1.29 – 1.80 | **<0.001** |
| Family History of CAD | | 1.13 | 0.96 – 1.32 | 0.137 | - | - | **-** |
| Peripheral Vascular Disease | | 1.27 | 0.85 – 1.90 | 0.238 | - | - | - |
| Previous CAD | | 1.85 | 1.61 – 2.12 | **<0.001** | 0.86 | 0.60 – 1.22 | 0.388 |
| Previous MI | | 1.71 | 1.46 – 2.01 | **<0.001** | 0.88 | 0.69 – 1.12 | 0.297 |
| Previous PCI | | 1.75 | 1.51 – 2.01 | **<0.001** | 1.27 | 0.93 – 1.72 | 0.135 |
| Previous CABG | | 2.83 | 2.27 – 3.53 | **<0.001** | 1.47 | 1.10 – 1.97 | **0.010** |
| Resting RWMA | | 4.47 | 3.80 – 5.25 | **<0.001** | 3.95 | 3.27 – 4.77 | **<0.001** |
|  | |  |  |  |  |  |  |

**Supplemental Table 1**: Univariate and multivariate logistic regression examining predictors of a positive stress echocardiogram. Statistically significant univariate predictors were included in the multivariate model.

|  | | **Positive SE**  **(N=1050)** | **Negative SE**  **(N=4453)** | ***P*-value^†^** | **Overall**  **(N=5503)** |
| --- | --- | --- | --- | --- | --- |
| Exercise Stress Echo (%) | | 303/1050 (28.9) | 1645/4451 (37.0) | **<0.001** | 1948/5501 (35.4) |
|  | Treadmill (%)* | 237/301 (78.7) | 1280/1621 (79.0) | 0.930 | 1517/1922 (78.9) |
|  | Bicycle (%)* | 63/301 (20.9) | 341/2621 (13.0) | 0.967 | 404/1922 (21.0) |
| Dobutamine Stress Echo (%) | | 730/1050 (69.5) | 2790/4451 (62.7) | **<0.001** | 3520/5501 (64.0) |
|  | Maximum Dobutamine Dose (mcg/kg/min), median (IQR) | 30 (20 – 30) | 30 (20 – 30) | 0.054 | 30 (20 – 30) |
| Atropine Use (%) | | 354/1047 (33.8) | 1213/4430 (27.4) | **<0.001** | 1567/5477 (28.6) |
| Paced Stress Echo (%) | | 17/1050 (1.6) | 13/4451 (<0.1) | **<0.001** | 30/5501 (0.5) |
| Dipyridamole Stress Echo (%) | | 0/1050 (0.0) | 3/4451 (<0.1) | - | 3/5501 (<0.1) |
| Contrast Use (%) | | 915/1045 (87.6) | 3395/4410 (77.0) | **<0.001** | 4310/5455 (79.0) |
| Resting Rhythm | |  |  |  |  |
|  | Sinus Rhythm (%) | 660/732 (90.2) | 3470/3699 (93.8) | **0.019** | 4130/4431 (93.2) |
|  | Atrial Fibrillation/Flutter (%) | 38/732 (5.2) | 125/3699 (3.4) | **0.17** | 163/4431 (3.7) |
|  | Paced (%) | 0/732 (0.0) | 11/3699 (0.3) | 0.679 | 11/4431 (0.2) |
|  | Other Rhythm (Not Specified) (%) | 34/732 (4.6) | 93/3699 (2.5) | **0.002** | 127/4431 (2.9) |
| Resting RWMA Present (%) | | 347 (33.0) | 443 (9.9) | **<0.001** | 790/5503 (14.4) |
| Ischaemic Burden (number of segments), median (IQR) | | 2 (2 – 4) | - | - | - |
|  | 1 – 2 Segments (%) | 489/972 (46.6) | - | - | - |
|  | 3 – 4 Segments (%) | 271/972 (25.8) | - | - | - |
|  | 5+ Segments (%) | 212/972 (20.2) | - | - | - |
|  |  |  |  |  |  |

**Supplemental Table 2:** Stress Echocardiogram Performance

**Supplemental Table 2**: Stress echocardiogram performance data. Presented as n./total n. (percentage). **^†^***P*-value for comparison between positive and negative stress echocardiogram. *Percentages given for treadmill and bicycle exercise relate to proportion of exercise stress echocardiograms.

**Supplemental Table 3:** Cohort Demographics Compared with Previous EVAREST Cohort

|  | | **Long-Term**  **Follow-Up**  **Cohort**  **(N=5503)** | **EVAREST**  **Cohort**  **From ref [9]**  **(N=5131)** | ***P*-value^†^** |
| --- | --- | --- | --- | --- |
| Male (%) | | 3238/5503 (58.8) | 2823/5131 (55.0) | **<0.001** |
| Age (years), median (IQR) | | 66 (57 – 74) | 66 (57 – 74) | 0.688 |
| BMI (Kg/m^2)^, median (IQR) | | 28.0 (24.9 – 31.7) | 28.3 (25.1 – 35.8) | 0.156 |
| BSA (m^2^), median (IQR) | | 1.95 (1.79 – 2.12) | 1.94 (1.78 – 2.10) | **0.007** |
| Smoking Status | |  |  |  |
|  | Non-Smoker (%) | 2656/5327 (49.9) | 2525/4952 (51.0) | 0.252 |
|  | Ex-Smoker (%) | 2033/5327 (38.2) | 1783/4952 (36.0) | **0.024** |
|  | Current Smoker (%) | 638/5327 (12.0) | 644/4952 (13.0) | 0.115 |
| Hypertension (%) | | 2951/5344 (55.2) | 2197/4947 (44.4) | **<0.001** |
| Hypercholesterolaemia (%) | | 2485/5344 (46.5) | 1841/4947 (37.2) | **<0.001** |
| Diabetes Mellitus (%) | | 1168/5344 (21.9) | 853/4947 (17.2) | **<0.001** |
| Peripheral Vascular Disease (%) | | 138/5345 (2.6) | 157/4947 (3.2) | 0.072 |
| Family History of Coronary Disease (%) | | 1204/5344 (22.5) | 72/4947 (1.5) | **<0.001** |
| Pre-existing Coronary Artery Disease (%) | | 1862/5483 (34.0) | 1868/5088 (36.7) | **0.003** |
|  | Previous MI (%) | 1006/5472 (18.4) | 867/5047 (17.2) | 0.106 |
|  | Previous PCI (%) | 1483/5475 (27.1) | 1547/5056 (30.6) | **<0.001** |
|  | Previous CABG (%) | 373/5478 (6.8) | 387/5107 (7.6) | 0.126 |

**Supplemental Table 3:** Comparison of demographic data between the long-term follow-up cohort reported in this paper with the EVAREST cohort used to report six-month outcome data [9]. Presented as n./total n. (percentage). ^†^*P*-value for comparison between long-term follow-up cohort and six-month outcome cohort [9].

| **Supplemental Table 4: Cox Proportional Hazard Model – All-Cause Mortality and Cardiac-Related Mortality** | | | | | | | | |
| --- | --- | --- | --- | --- | --- | --- | --- | --- |
|  | | | Univariable | | | Multivariable | | |
|  | | | **HR** | **95% CI** | ***P*-value** | **HR** | **95% CI** | ***P*-value** |
| All-Cause Mortality | Age | | 1.08 | 1.07 – 1.10 | **<0.001** | 1.09 | 1.07 – 1.10 | **<0.001** |
|  | Sex (Male) | | 2.00 | 1.50 – 2.67 | **<0.001** | 1.72 | 1.27 – 2.34 | **<0.001** |
|  | Smoking Status | |  |  |  |  |  |  |
|  |  | Ex-Smoker | 1.83 | 1.37 – 2.45 | **<0.001** | 1.60 | 1.19 – 2.15 | **0.002** |
|  |  | Current Smoker | 2.69 | 1.84 – 3.92 | **<0.001** | 3.67 | 2.50 – 5.40 | **<0.001** |
|  | Hypertension | | 1.51 | 1.15 - 1.98 | **0.003** | 0.99 | 0.75 – 1.32 | 0.965 |
|  | Hypercholesterolaemia | | 1.23 | 0.95 – 1.59 | 0.117 | - | - | - |
|  | Diabetes | | 2.12 | 1.63 – 2.77 | **<0.001** | 1.96 | 1.49 – 2.59 | **<0.001** |
|  | Previous Coronary Disease | | 1.29 | 0.99 – 1.67 | 0.058 | - | - | - |
|  | Resting RWMA | | 2.00 | 1.49 – 2.68 | **<0.001** | 1.41 | 1.02 – 1.95 | **0.039** |
|  | Positive SE | | 1.55 | 1.16 – 2.07 | **0.003** | 0.96 | 0.68 – 1.35 | 0.813 |
|  | Elective Revascularisation | | 1.82 | 1.21 – 2.73 | **0.004** | 1.33 | 0.85 – 2.09 | 0.220 |
|  | | | | | | | | |
| Cardiac-Related Mortality | Age | | 1.09 | 1.06 – 1.12 | **<0.001** | 1.09 | 1.06 – 1.13 | **<0.001** |
|  | Sex (Male) | | 2.14 | 1.22 – 3.78 | **0.008** | 1.26 | 0.70 – 2.28 | 0.446 |
|  | Smoking Status | |  |  |  |  |  |  |
|  |  | Ex-Smoker | 3.01 | 1.68 – 5.40 | **<0.001** | 2.60 | 1.42 – 4.62 | **0.002** |
|  |  | Current Smoker | 2.85 | 1.26 – 6.47 | **0.012** | 3.73 | 1.62 – 8.58 | **0.002** |
|  | Hypertension | | 1.31 | 0.79 – 2.17 | 0.299 | - | - | - |
|  | Hypercholesterolaemia | | 1.32 | 0.81 – 2.16 | 0.270 | - | - | - |
|  | Diabetes | | 3.94 | 2.41 – 6.44 | **<0.001** | 3.26 | 1.97 – 5.40 | **<0.001** |
|  | Previous Coronary Disease | | 3.20 | 1.92 – 5.34 | **<0.001** | 1.61 | 0.92 – 2.81 | 0.098 |
|  | Resting RWMA | | 4.32 | 2.63 – 7.10 | **<0.001** | 2.25 | 1.26 – 4.01 | **0.006** |
|  | Positive SE | | 2.52 | 1.51 – 4.20 | **<0.001** | 1.20 | 0.68 – 2.12 | 0.520 |
|  | Elective Revascularisation | | 2.08 | 0.99 – 4.36 | 0.053 | - | - | - |

**Supplemental Table 4:** Cox proportional hazard model for all-cause mortality (top) and cardiac-related mortality (bottom). Statistically significant univariable predictors were included in the multivariable model.

| **Supplemental Table 5: Cox Proportional Hazard Model - Myocardial Infarction** | | | | | | | | | |
| --- | --- | --- | --- | --- | --- | --- | --- | --- | --- |
|  | | | | Univariable | | | Multivariable | | |
|  | | | | **HR** | **95% CI** | ***P*-value** | **HR** | **95% CI** | ***P*-value** |
| Stress Echo Outcome | Age | | | 1.03 | 1.01 – 1.05 | **<0.001** | 1.03 | 1.01 – 1.05 | **0.015** |
|  | Sex (Male) | | | 2.22 | 1.37 – 3.58 | **0.001** | 1.64 | 0.99 – 2.69 | 0.052 |
|  | Smoking Status | | |  |  |  |  |  |  |
|  |  | | Ex-Smoker | 1.33 | 0.85 – 2.09 | 0.206 | - | - | - |
|  |  | | Current Smoker | 1.37 | 0.72 – 2.63 | 0.338 | - | - | - |
|  | Hypertension | | | 1.31 | 0.85 – 2.01 | 0.216 | - | - | - |
|  | Hypercholesterolaemia | | | 1.04 | 0.69 – 1.58 | 0.849 | - | - | - |
|  | Diabetes | | | 2.03 | 1.32 – 3.13 | **0.001** | 1.57 | 1.01 – 2.44 | **0.045** |
|  | Previous Coronary Disease | | | 2.13 | 1.41 – 3.22 | **<0.001** | 1.35 | 0.85 – 2.12 | 0.201 |
|  | Resting RWMA | | | 3.12 | 2.02 – 4.81 | **<0.001** | 1.74 | 1.07 – 2.84 | **0.027** |
|  | Positive SE | | | 3.95 | 2.62 – 5.96 | **<0.001** | 2.71 | 1.73 – 4.24 | **<0.001** |
|  | Elective Revascularisation | | | 1.12 | 0.49 – 2.57 | 0.783 | - | - | **-** |
|  | | | | | | | | | |
| Degree of Ischaemia | Age | | | 1.03 | 1.01 – 1.05 | **<0.001** | 1.03 | 1.01 – 1.05 | **0.009** |
|  | Sex (Male) | | | 2.22 | 1.37 – 3.58 | **0.001** | 1.65 | 0.99 – 2.75 | 0.054 |
|  | Smoking Status | | |  |  |  |  |  |  |
|  |  | | Ex-Smoker | 1.33 | 0.85 – 2.09 | 0.206 | - | - | - |
|  |  | | Current Smoker | 1.37 | 0.72 – 2.63 | 0.338 | - | - | - |
|  | Hypertension | | | 1.31 | 0.85 – 2.01 | 0.216 | - | - | - |
|  | Hypercholesterolaemia | | | 1.04 | 0.69 – 1.58 | 0.849 | - | - | - |
|  | Diabetes | | | 2.03 | 1.32 – 3.13 | **0.001** | 1.48 | 0.93 – 2.34 | 0.095 |
|  | Previous Coronary Disease | | | 2.13 | 1.41 – 3.22 | **<0.001** | 1.37 | 0.86 – 2.19 | 0.183 |
|  | Resting RWMA | | | 3.12 | 2.02 – 4.81 | **<0.001** | 1.49 | 0.89 – 2.50 | 0.130 |
|  | Ischaemic Burden | | |  |  |  |  |  |  |
|  |  | 1 – 2 Segments | | 2.72 | 1.50 – 4.94 | **<0.001** | 2.12 | 1.15 – 3.91 | **0.017** |
|  |  | 3 – 4 Segments | | 3.94 | 2.05 – 7.59 | **<0.001** | 2.47 | 1.22 – 5.02 | **0.012** |
|  |  | 5+ Segments | | 5.92 | 3.21 – 10.92 | **<0.001** | 4.23 | 2.23 – 8.03 | **<0.001** |
|  | Elective Revascularisation | | | 1.12 | 0.49 – 2.57 | **0.783** | - | - | **-** |

**Supplemental Table 5:** Cox proportional hazard models for myocardial infarction. Statistically significant univariable predictors were included in a multivariable model to calculate adjusted hazard ratios. Two models were constructed, one examining overall stress echocardiogram result (top), the second examining impact of degree of ischaemia on hazard ratios (bottom).

| **Supplemental Table 6: Cox Proportional Hazard Model - Revascularisation** | | | | | | | | |
| --- | --- | --- | --- | --- | --- | --- | --- | --- |
|  | | | Univariable | | | Multivariable | | |
|  | | | **HR** | **95% CI** | ***P*-value** | **HR** | **95% CI** | ***P*-value** |
| Stress Echo Outcome | Age | | 1.02 | 1.01 – 1.02 | **0.002** | 1.00 | 0.99 – 1.01 | 0.626 |
|  | Sex (Male) | | 2.31 | 1.82 – 2.94 | **<0.001** | 1.77 | 1.37 – 2.27 | **<0.001** |
|  | Smoking Status | |  |  |  |  |  |  |
|  |  | Ex-Smoker | 1.13 | 0.90 – 1.41 | 0.292 | - | - | - |
|  |  | Current Smoker | 1.29 | 0.93 – 1.77 | 0.123 | - | - | - |
|  | Hypertension | | 1.65 | 1.32 – 2.05 | **<0.001** | 1.18 | 0.94 – 1.49 | 0.160 |
|  | Hypercholesterolaemia | | 1.56 | 1.28 – 1.94 | **<0.001** | 1.08 | 0.87 – 1.34 | 0.499 |
|  | Diabetes | | 1.96 | 1.58 – 2.44 | **<0.001** | 1.33 | 1.06 – 1.66 | **0.015** |
|  | Previous Coronary Disease | | 1.77 | 1.44 – 2.18 | **<0.001** | 1.00 | 0.80 – 1.26 | 0.992 |
|  | Resting RWMA | | 2.92 | 2.35 – 3.65 | **<0.001** | 1.20 | 0.94 – 1.53 | 0.139 |
|  | Positive SE | | 12.58 | 10.02 – 15.80 | **<0.001** | 10.52 | 8.26 – 13.41 | **<0.001** |
|  | | | | | | | | |
| Degree of Ischaemia | Age | | 1.02 | 1.01 – 1.02 | **0.002** | 1.00 | 0.99 – 1.01 | 0.803 |
|  | Sex (Male) | | 2.31 | 1.82 – 2.94 | **<0.001** | 1.76 | 1.36 – 2.29 | **<0.001** |
|  | Smoking Status | |  |  |  |  |  |  |
|  |  | Ex-Smoker | 1.13 | 0.90 – 1.41 | 0.292 | - | - | - |
|  |  | Current Smoker | 1.29 | 0.93 – 1.77 | 0.123 | - | - | - |
|  | Hypertension | | 1.65 | 1.32 – 2.05 | **<0.001** | 1.19 | 0.94 – 1.52 | 0.150 |
|  | Hypercholesterolaemia | | 1.56 | 1.28 – 1.94 | **<0.001** | 1.03 | 0.82 – 1.30 | 0.791 |
|  | Diabetes | | 1.96 | 1.58 – 2.44 | **<0.001** | 1.20 | 0.94 – 1.52 | 0.139 |
|  | Previous Coronary Disease | | 1.77 | 1.44 – 2.18 | **<0.001** | 1.12 | 0.88 – 1.41 | 0.370 |
|  | Resting RWMA | | 2.92 | 2.35 – 3.65 | **<0.001** | 1.09 | 0.84 – 1.40 | 0.524 |
|  | Ischaemic Burden | |  |  |  |  |  |  |
|  |  | 1 – 2 Segments | 8.22 | 6.17 – 10.95 | **<0.001** | 7.30 | 5.42 – 9.83 | **<0.001** |
|  |  | 3 – 4 Segments | 11.09 | 8.07 – 15.25 | **<0.001** | 9.08 | 6.49 – 12.71 | **<0.001** |
|  |  | 5+ Segments | 27.01 | 20.43 – 35.70 | **<0.001** | 22.79 | 16.92 – 30.69 | **<0.001** |

**Supplemental Table 6:** Cox proportional hazard models for revascularisation. Statistically significant univariable predictors were included in a multivariable model to calculate adjusted hazard ratios. Two models were constructed, one examining overall stress echocardiogram result (top), the second examining impact of degree of ischaemia on hazard ratios (bottom).

| **Supplemental Table 7: Cox Proportional Hazard Model - Composite Endpoint** | | | | | | | | |
| --- | --- | --- | --- | --- | --- | --- | --- | --- |
|  | | | Univariable | | | Multivariable | | |
|  | | | **HR** | **95% CI** | ***P*-value** | **HR** | **95% CI** | ***P*-value** |
| Stress Echo Outcome | Age | | 1.05 | 1.03 – 1.07 | **<0.001** | 1.05 | 1.03 – 1.06 | **<0.001** |
|  | Sex (Male) | | 2.14 | 1.47 – 3.12 | **<0.001** | 1.52 | 1.02 – 2.26 | **0.041** |
|  | Smoking Status | |  |  |  |  |  |  |
|  |  | Ex-Smoker | 1.69 | 1.18 – 2.42 | **0.005** | 1.38 | 0.96 – 1.99 | 0.085 |
|  |  | Current Smoker | 1.86 | 1.12 – 3.09 | **0.017** | 1.85 | 1.10 – 3.12 | **0.021** |
|  | Hypertension | | 1.31 | 0.93 – 1.84 | 0.121 | - | - | - |
|  | Hypercholesterolaemia | | 1.13 | 0.81 – 1.57 | 0.464 | - | - | - |
|  | Diabetes | | 2.53 | 1.81 – 3.53 | **<0.001** | 2.04 | 1.44 – 2.87 | **<0.001** |
|  | Previous Coronary Disease | | 2.35 | 1.69 – 3.26 | **<0.001** | 1.34 | 0.93 – 1.93 | 0.118 |
|  | Resting RWMA | | 3.54 | 2.52 – 4.97 | **<0.001** | 1.93 | 1.30 – 2.86 | **0.001** |
|  | Positive SE | | 3.30 | 2.37 – 4.59 | **<0.001** | 2.03 | 1.41 – 2.93 | **<0.001** |
|  | Elective Revascularisation | | 1.53 | 0.86 – 2.70 | 0.147 | - | - | **-** |
|  | | | | | | | | |
| Degree of Ischaemia | Age | | 1.05 | 1.03 – 1.07 | **<0.001** | 1.05 | 1.03 – 1.07 | **<0.001** |
|  | Sex (Male) | | 2.14 | 1.47 – 3.12 | **<0.001** | 1.50 | 0.99 – 2.25 | 0.051 |
|  | Smoking Status | |  |  |  |  |  |  |
|  |  | Ex-Smoker | 1.69 | 1.18 – 2.42 | **0.005** | 1.44 | 0.99 – 2.11 | 0.060 |
|  |  | Current Smoker | 1.86 | 1.12 – 3.09 | **0.017** | 1.97 | 1.15 – 3.38 | **0.014** |
|  | Hypertension | | 1.31 | 0.93 – 1.84 | 0.121 | - | - | - |
|  | Hypercholesterolaemia | | 1.13 | 0.81 – 1.57 | 0.464 | - | - | - |
|  | Diabetes | | 2.53 | 1.81 – 3.53 | **<0.001** | 1.97 | 1.38 – 2.82 | **<0.001** |
|  | Previous Coronary Disease | | 2.35 | 1.69 – 3.26 | **<0.001** | 1.33 | 0.91 – 1.94 | 0.136 |
|  | Resting RWMA | | 3.54 | 2.52 – 4.97 | **<0.001** | 1.70 | 1.12 – 2.57 | **0.012** |
|  | Ischaemic Burden | |  |  |  |  |  |  |
|  |  | 1 – 2 Segments | 2.26 | 1.39 – 3.68 | **0.001** | 1.63 | 0.98 – 2.70 | 0.059 |
|  |  | 3 – 4 Segments | 3.64 | 2.16 – 6.13 | **<0.001** | 1.95 | 1.09 – 3.46 | **0.024** |
|  |  | 5+ Segments | 4.34 | 2.54 – 7.41 | **<0.001** | 2.77 | 1.59 – 4.85 | **<0.001** |
|  | Elective Revascularisastion | | 1.53 | 0.86 – 2.70 | 0.147 | - | - | **-** |

**Supplemental Table 7:** Cox proportional hazard models for composite endpoint (myocardial infarction or cardiac-related mortality). Statistically significant univariable predictors were included in a multivariable model to calculate adjusted hazard ratios. Two models were constructed, one examining overall stress echocardiogram result (top), the second examining impact of degree of ischaemia on hazard ratios (bottom).
